# EBV, HCMV, HHV6, and HHV7 Screening in Bone Marrow Samples from Children with Acute Lymphoblastic Leukemia

**DOI:** 10.1155/2014/548097

**Published:** 2014-09-18

**Authors:** A. Morales-Sánchez, E. N. Pompa-Mera, A. Fajardo-Gutiérrez, F. J. Alvarez-Rodríguez, V. C. Bekker-Méndez, J. de Diego Flores-Chapa, J. Flores-Lujano, E. Jiménez-Hernández, J. G. Peñaloza-González, M. C. Rodríguez-Zepeda, J. R. Torres-Nava, M. M. Velázquez-Aviña, R. Amador-Sánchez, M. Alvarado-Ibarra, N. Reyes-Zepeda, R. M. Espinosa-Elizondo, M. L. Pérez-Saldivar, J. C. Núñez-Enríquez, J. M. Mejía-Aranguré, E. M. Fuentes-Pananá

**Affiliations:** ^1^Unidad de Investigación Médica en Epidemiología Clínica, Hospital de Pediatría, CMN Siglo-XXI, Instituto Mexicano del Seguro Social (IMSS), Avenida Cuauhtémoc 330, Colonia Doctores, 06720 México, DF, Mexico; ^2^Unidad de Investigación en Virología y Cáncer, Hospital Infantil de México Federico Gómez, Dr. Márquez 162, Colonia Doctores, 06720 México, DF, Mexico; ^3^Facultad de Medicina, Universidad Nacional Autónoma de México, Avenida Universidad 3000, 04510 Mexico City, DF, Mexico; ^4^Unidad de Investigación Médica en Inmunología e Infectología, Hospital de Infectología, CMN La Raza, IMSS, Calzada Vallejo y Jacarandas S/N Colonia La Raza, 02990 Mexico City, DF, Mexico; ^5^Grupo Mexicano Interinstitucional para la Identificación de las Causas de la Leucemia Infantil (GMIICLI), Avenida Cuauhtémoc 330, Colonia Doctores, 06720 Mexico City, DF, Mexico; ^6^Servicio de Oncología, Hospital Pediátrico Moctezuma, Secretaría de Salud (SSa) del D.F., Oriente 158-189, Colonia Moctezuma 2a Sección, 15530 Mexico City, DF, Mexico; ^7^Servicio de Hematología Pediátrica, CMN 20 de Noviembre, Instituto de Seguridad Social al Servicio de los Trabajadores del Estado, Félix Cuevas 540, Colonia Del Valle, 03229 Mexico City, DF, Mexico; ^8^Servicio de Hematología Pediátrica, Hospital General Gaudencio González Garza, CMN La Raza, IMSS, Calzada Vallejo y Jacarandas S/N Colonia La Raza, 02990 Mexico City, DF, Mexico; ^9^Servicio de Onco-Pediatría, Hospital Juárez de México, SSa, Avenida Instituto Politécnico Nacional 5160, Colonia Magdalena de las Salinas, 07760 Mexico City, DF, Mexico; ^10^Servicio de Hematología, UMAE, Hospital de Pediatría, CMN Siglo-XXI, IMSS, Avenida Cuauhtémoc 330, Colonia Doctores, 06720 Mexico City, DF, Mexico; ^11^Hospital General Regional Carlos McGregor Sánchez Navarro, IMSS, Avenida Gabriel Mancera 222, Colonia Del Valle, 03100 Mexico City, DF, Mexico; ^12^Servicio de Hematología Pediátrica, Hospital General de México, SSa, Eje 2A Sur (Dr. Balmis) 148, Colonia Doctores, 06726 Mexico City, DF, Mexico

## Abstract

Acute lymphoblastic leukemia (ALL) is the most common cancer in childhood worldwide and Mexico has reported one of the highest incidence rates. An infectious etiology has been suggested and supported by epidemiological evidences; however, the identity of the involved agent(s) is not known. We considered that early transmitted lymphotropic herpes viruses were good candidates, since transforming mechanisms have been described for them and some are already associated with human cancers. In this study we interrogated the direct role of EBV, HCMV, HHV6, and HHV7 human herpes viruses in childhood ALL. Viral genomes were screened in 70 bone marrow samples from ALL patients through standard and a more sensitive nested PCR. Positive samples were detected only by nested PCR indicating a low level of infection. Our result argues that viral genomes were not present in all leukemic cells, and, hence, infection most likely was not part of the initial genetic lesions leading to ALL. The high statistical power of the study suggested that these agents are not involved in the genesis of ALL in Mexican children. Additional analysis showed that detected infections or coinfections were not associated with prognosis.

## 1. Introduction

Acute lymphoblastic leukemia (ALL) is the most common childhood cancer worldwide and Mexico has reported one of the highest ALL incidence rates [1, 2]. While new therapies have notably improved ALL outcome in recent years [3], pathogenic events leading to disease development remain largely unknown [4]. An infectious etiology has been suggested by different hypothesis favoring either direct or indirect mechanisms of transformation [5–7]. For infectious agents, direct oncogenic mechanisms refer to expression of viral oncogenes together with deregulation of cellular oncogenes and/or tumor suppressor genes. Indirect mechanisms are mainly triggered by an inflammatory milieu with production of mutagenic molecules or immunosuppression with loss of the cancer immune surveillance mechanisms [8]. The former mode of transformation implies that the infectious agent acts from within the cell and thus, after the cancer clonal expansion, it is carried in all tumor cells, as it has been documented for tumor herpes viruses [9, 10]; while indirectly acting infectious carcinogenic agents do not necessarily infect the cell forming the tumor.

The* Delayed infection *hypothesis by Greaves proposes that exposure to infectious agents very early in life protects from ALL as it modulates maturation of the immune system. However, late exposures lead to an aberrant immune response promoting leukemogenesis by an indirect mechanism [5]. On the opposite side, Smith proposes that oncogenic viruses transmitted during intrauterine life or the first year of life are able to infect immature lymphocytes and promote leukemia through a direct transformation mechanism [7]. Epidemiological, clinical, and molecular evidence have been searched to support either direct or indirect mechanisms with variable and even opposite results (reviewed in [11]). We considered that the high incidence of childhood ALL in Mexico City was more closely correlated with high incidence of early infection of a developing country [1]. In agreement, we recently showed that serious infections requiring hospitalization in the first year of life were associated with increased risk of ALL in children with Down syndrome [12], better supporting the notion that early infections can promote pediatric leukemia as proposed by the Smith's hypothesis.

In this study, we explored the idea that viruses from which oncogenic capacities have been documented may promote leukemogenesis through a direct transforming mechanism. We selected members of the herpesviridae family, Epstein Barr virus (EBV), human cytomegalovirus (HCMV), and human herpes virus 6 (HHV6A and B) and 7 (HHV7) because they are lymphotropic viruses often transmitted in the first months of life. EBV is a potent transforming agent and has been consistently associated with several human malignancies including pediatric lymphomas [10]. Although, HCMV and HHV6 are not considered carcinogenic, their transforming ability has been shown in* in vitro* studies [13–16]. Additionally, HCMV has been defined as oncomodulator because of its ability to infect tumor cells and alter proliferation, survival, angiogenesis, and invasiveness increasing the tumor aggressiveness [17, 18]. HHV6 has previously been associated with several hematological malignances, including childhood acute leukemia, through serological case-control studies, although with heterogeneous results [19, 20]. An HHV7 transforming role has not been shown; however, there are proposals about its role as cofactor in T-cell and B-cell lymphomas [21, 22]. Moreover, HHV7 may potentiate the pathogenic role of other herpes viruses [23, 24]. We assessed whether EBV, HCMV, HHV6, and HHV7 were involved in the genesis of childhood B-cell and T-cell ALL through a direct transformation mechanism. ALL bone marrow samples were tested by two PCRs with different detection limits. Considering that viruses acting through direct transforming mechanisms behave like driver genetic lesions that are preserved throughout tumor development, we designed a PCR test to equate the number of infected cells with the number of tumor cells and an even more sensitive PCR to detect evidence of infection. We found that less than 20% of the samples were positive by at least one of the viruses tested. Because positive samples showed low infection levels, these data do not support a direct role for EBV, HCMV, HHV6, and HHV7 in the genesis of pediatric ALL from Mexican children. Further analysis of the positives samples showed no association between detected infections or coinfections and prognosis.

## 2. Materials and Methods

### 2.1. Ethics Statement

This study was approved by the ethical and scientific review boards of the Mexican Institute of Social Security (IMSS): the National Commission of Scientific Research and the Ethics Committee on Research. Prior to sample collection, parents of enrolled patients were informed on the nature of the study and those who were willing to participate signed a letter of consent; children older than 10 years also signed a letter of assent. All patients enrolled were treated according to the ethical guidelines of our institution.

### 2.2. Patients and Biological Samples

The cases recruited in this study belong to the Mexican Inter-institutional Group for the Identification of the Causes of Childhood Leukaemia (MIGICCL; Mexico City, Mexico), a member of the Childhood Leukemia International Consortium (CLIC) since 2012. During the period of the study (January 1, 2010, to August 30, 2012) there were 553 patients diagnosed with B- or T-cell ALL; however, there were sufficient bone marrow samples from only 70 pretreatment patients (66 from B-cell ALL and 4 from T-cell ALL) to include in the present study (Table 1). Two mL of bone marrow were collected in 0.1 M sodium citrate solution (TEKNOVA, Hollister CA, USA) from the included patients and mononuclear cells were isolated by a density gradient centrifugation on Histopaque-1077 (Sigma-Aldrich, St. Luis, NO).

### 2.3. Control Cell Lines and Plasmid DNA

For EBV detection, Raji (ATCC, CCL-86) and Ramos (ATCC, CRL-1596) cell lines were used as positive and negative controls, respectively. Both cell lines were cultured in advanced RPMI medium supplemented with 4% of fetal bovine serum and 1X hepes (all from Gibco, Carlsbad, CA) and maintained at 37°C in 5% of CO_2_. Control plasmids were constructed for HCMV, HHV6, and HHV7 detection. Purified HCMV viral genome was kindly provided by Dr. M. Ruiz-Tachiquin (Pediatric Hospital at the CMN SXXI, IMSS, Mexico City, Mexico). HHV6 DNA was obtained from MOLT3 cell line (ATCC CRL-1552) infected with viral particles of HHV6 Z29 strain (ATCC VR-1467). HHV7 DNA was purified from infected lymphocytes kindly provided by Dr. E. Sevilla (National Institute of Respiratory Diseases, Mexico City, Mexico). A fragment of HCMV UL83, HHV6 U94, and HHV7 U42 genes were PCR amplified using primers described in Table 2 (standard PCR) and cloned into T-easy pGEM plasmid (Promega, Madison, WI) according to manufacturer's instructions. The identity of cloned products was confirmed by sequencing.

### 2.4. DNA Purification

DNA was purified from bone marrow mononuclear cells or control cell lines using QIAamp DNA extraction kits (Qiagen, Hilden, Germany) according to the manufacturer's instructions. Plasmid DNA was purified using PureLink Quick Plasmid Miniprep Kit (Invitrogen, Carlsbad, CA) following the manufacturer's instruction. Purified DNA was quantified using a spectrophotometer NanoDrop 1000 (Thermo Fisher Scientific, Waltham, MA). The DNA quality and integrity were evaluated through electrophoretic landslide, optical density (260/280 ratio), and amplification of *β*-actin endogenous gene (genomic DNA).

### 2.5. Standard and Nested PCRs

Standard and nested PCR mix contained 1x Taq Polymerase buffer, 1.5 to 2.5 mM MgCl_2_ (MgCl_2_ concentration was optimized for every PCR), 200 *μ*M deoxynucleotide triphosphate (dNTP), 2.5 U of Taq Polymerase (all from Thermo Fisher Scientific, Waltham, MA), and 200 nM of each primer (IDT Technologies). For standard PCR, 100 ng of DNA from samples or control cell lines or 5.5 × 10^−5^ ng from control plasmid (equivalent to the number of moles contained in 100 ng of genomic DNA) were used. Plasmids were linearized with restriction enzyme NdeI (New England BioLabs) before use and mixed with DNA from Daudi cell line in order to run the amplification reaction under identical mass/volume DNA concentration for both samples and controls. For the nested PCR, 0.5 or 0.05 *μ*L (1 : 100 or 1 : 1000 dilution resp.) of product of first round PCR was used as template. All PCR reactions were carried out in a final volume of 50 *μ*L. All primer sequences and cycling conditions used are detailed in Table 2. PCR products were analyzed by electrophoresis in 1.8% agarose gels stained with ethidium bromide and photographed under ultraviolet light using the Quantum ST4 System (VilberLourmat, Torcy, Marne-la-Vallée, FR). The viral identity of PCR amplified fragments was confirmed by sequencing.

### 2.6. Nucleotide Sequencing

The cloned viral gene fragments and PCR amplified fragments were excised from 1% agarose gels and purified using QIAquick gel extraction kit (Qiagen Hilden, Germany), following the manufacturer's instructions. Direct automated sequencing of both forward and reverse strands was carried out using the BigDye Terminator Cycle Sequencing kit (Applied Biosystems, Foster City, CA). About 20 ng of template DNA was added for each reaction; the program included 25 cycles as described: denaturation at 95°C for 30 sec, annealing at 50°C for 15 sec, and extension at 60°C for 4 min. Samples were analyzed in an ABI Prism 3130 Genetic Analyzer (Applied Biosystems, Foster City, CA). The obtained sequences were examined using the Nucleotide BLAST program [25].

### 2.7. Statistical Analysis

The statistical power of the study was calculated considering the sample size (*N* = 70) and the probability to detect viral genomes supporting a direct transformation mechanism in at least one patient. The hypothetical frequencies of infection were 10% and 5%. The statistical package used for this analysis was Epi Info version 7.1.4 (Centers for Disease Control and Prevention, http://wwwn.cdc.gov/epiinfo/). Odds ratios with 95% confidence intervals were calculated to identify if the detected infections were associated with high-risk leukemia, relapse, or death. Mann-Whitney *U* test was used to compare age in months and leukocyte count with infection. The statistical package used for these analyses was Epidat version 3.0.

## 3. Results

### 3.1. Study Population

The study included 70 pretreatment children diagnosed with ALL, 66 were B-cell ALL and 4 were T-cell ALL. The diagnosis of ALL fulfilled the morphological and phenotypic criteria, including that at least 25% of the cells from the bone marrow sample were leukemic blasts. Demographic and clinic characteristics of the patients were extracted from medical records and are presented in Table 1.

### 3.2. PCRs Limit of Detection

**Figure 1:**
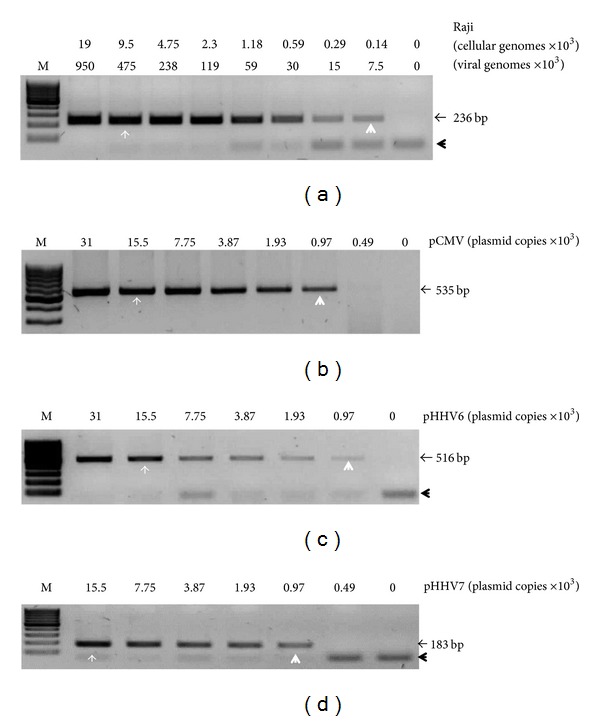
The PCRs limit of detection. Serial dilutions of DNA from control cell line and plasmid were PCR amplified in order to know the lower limit of detection of our screening test. For EBV (a), the limit of detection is expressed in number of cellular and viral genomes (from Raji cell line). For HCMV (b), HHV6 (c), and HHV7 (d) the limits of detection are expressed as plasmid copies. Lower limits of detection are indicated with a white arrowhead. White arrows point to the number of cells or plasmid copies that were used for viral detection in ALL samples. The sizes of PCR products are indicated. Black arrowheads point to residual primers.

To know the limit of detection of the standard PCRs, we first carried out amplification reactions using serial dilutions of DNA from control cell lines and plasmids (Figure 1). For EBV detection, we employed Raji cell line, which derives from an EBV-associated lymphoma and carries about 50 viral episomes per cell [26]. The amplification target was the BamHI W fragment, which is eleven times repeated in the EBV genome. The lower limit of detection of our reaction was of 7.5 × 10^3^ viral genomes (Figure 1(a)). We decided to use 9.5 × 10^3^ cells from bone marrow samples for EBV detection because we speculated that ALL samples might have similar frequencies to EBV episomes than those found in EBV-associated lymphomas. Still, if the number of viral genomes in leukemic cells were as low as the minimum reported for an EBV-associated cancer, namely, 7 viral genomes per cell in EBV-associated nasopharyngeal carcinoma [27], our test would be able to detect positive samples in as low as 25% of blasts (Figure 1(a)). For HCMV, HHV6, and HHV7 detection, we used plasmid DNA as control (see Material and Methods section). The limit of detection was about 0.97 × 10^3^ plasmid copies for every reaction (Figures 1(b), 1(c), and 1(d)). As we did not know the number of HCMV, HHV6, and HHV7 genomes that could be present in each leukemic blast, DNA from control plasmid and samples were used at equal molar concentration. We determined that using 15.5 × 10^3^ cells from samples (and 15.5 × 10^3^ plasmid copies mixed with DNA from 15.5 × 10^3^ negative cells as control) would be sufficient to detect up to one viral genome per cell even in samples with only 25% of leukemic blasts (Figures 1(b), 1(c), and 1(d)). We have previously used these PCRs in which the frequency of infected cells matches the frequency of cancer cells to address direct viral oncogenesis [28–30].

### 3.3. Viral Screening

**Figure 2:**
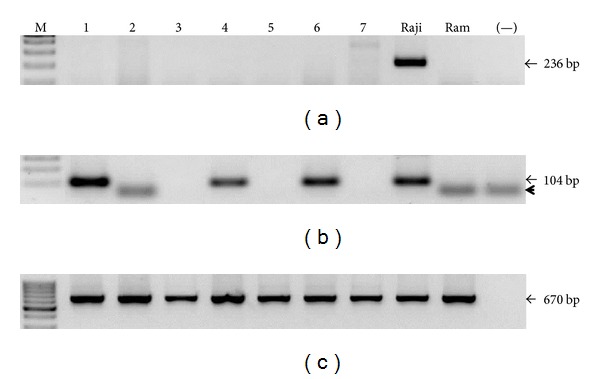
EBV screening. Image of seven representative samples showing that all of them were EBV negative by first round PCR (a) and positivity was only detected by nested PCR in 10 (14%; showing three) samples (b). Raji and Ramos (Ram) cell lines were used as positive and negative controls, respectively. A reaction without DNA was routinely run (−). (c) Amplification of *β* *-actin* endogenous gene. The sizes of PCR products are indicated. The black arrowhead points to residual primers.

**Figure 3:**
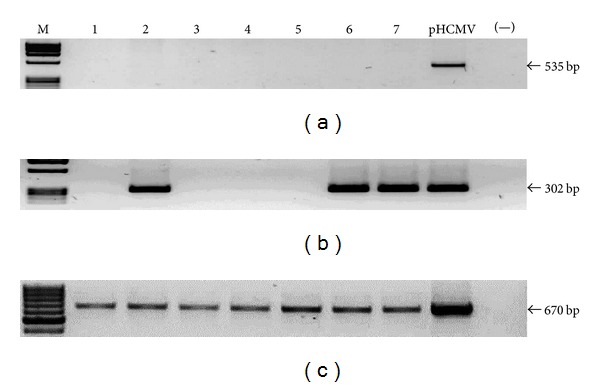
HCMV screening. Image of seven representative samples showing that all of them were HCMV negative by first round PCR (a) and positivity was only detected by nested PCR in 13 (19%, showing three) samples (b). Plasmid DNA was used as positive control (pHCMV). A reaction without DNA was routinely run (−). (c) Amplification of *β* *-actin* endogenous gene. The sizes of PCR products are indicated.

**Figure 4:**
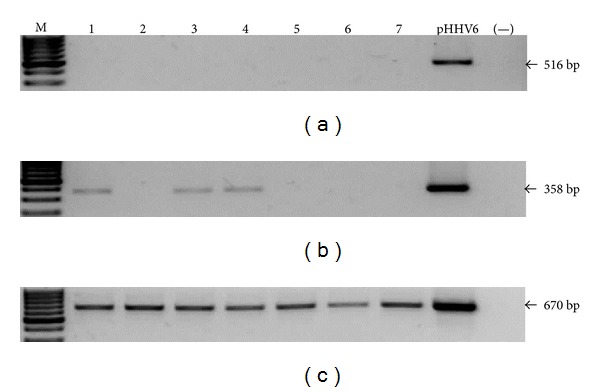
HHV6 screening. Image of seven representative samples showing that all of them were HHV6 negative by first round PCR (a) and positivity was only detected by nested PCR in 6 (9%, showing three) samples (b). Plasmid DNA was used as positive control (pHHV6). A reaction without DNA was routinely run (−). (c) Amplification of *β* *-actin* endogenous gene. The sizes of PCR products are indicated.

**Figure 5:**
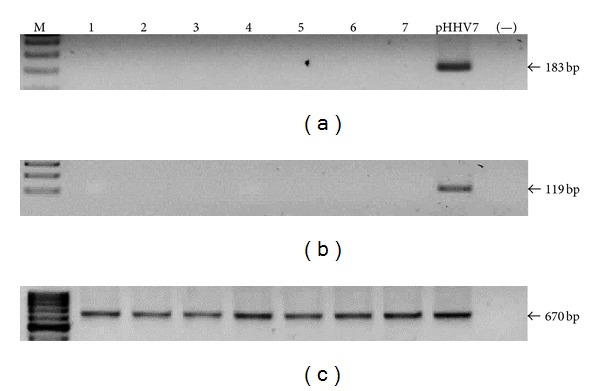
HHV7 screening. Image of seven representative samples showing that all of them were HHV7 negative by both first round PCR (a) and nested PCR (b). Plasmid DNA was used as positive control (pHHV7). A reaction without DNA was routinely run (−). (c) Amplification of *β* *-actin* endogenous gene. The sizes of PCR products are indicated.

Once the limit of detection was established for every reaction, we screened the presence of viral genomes in the samples. We do not observe any positive sample for any herpes virus tested by the first round PCR (Figures 2(a), 3(a), 4(a), and 5(a)). EBV, HCMV, and HHV6 positive samples were only detected through nested PCR in the following frequencies: 14%, 19%, and 9%, respectively, while HHV7 was not detected by this method (Figures 2(b), 3(b), 4(b), and 5(b)). Considering that direct carcinogenic viruses contribute to oncogenesis acting like driver genetic lesions, we would consider that if viral infection had a role in the transformation of the cell subjected to oncogenic clonal expansion, the progeny of that cell should all carry the viral genome. In that scenario, the viral genome should have been detected by the first PCR in agreement with the previously established limit of detection. All samples showed high DNA integrity (not shown) and the amplification of *β* *-actin* endogenous gene was positive (Figures 2(c), 3(c), 4(c), and 5(c)).

### 3.4. Power of the Study

We carried out a post hoc power analysis to calculate the probability that our results were true based in the obtained sample size and the frequency of the infection. The frequency of the infection was hypothetical because up to today none of the viruses studied have been shown to cause ALL. The statistical power test indicated that the probability to detect at least one positive subject (in the first PCR, supporting a direct transforming) from our study population (*N* = 70) was 99.99% and 95.00% from hypothetical frequencies of infection of 10% and 5%, respectively. Thus, our results suggest that EBV, HCMV, HHV6, and HHV7 are not involved in the genesis of childhood ALL in Mexican children.

### 3.5. Analysis of Infection and Prognosis

A possible role of the detected infections in prognosis was explored. Considering a previously documented probable interaction between the herpes viruses tested [31–35] we calculated the association between every infection or coinfection and the risk for high-risk leukemia, relapse, or death (Table 3). Coinfections included only double-infections: EBV+/HCMV+, EBV+/HHV6+, and HCMV+/HHV6+; triple infections were not detected. We did not find any significant association with this analysis (Table 3). We also compared the age (in months) at the moment of diagnosis and the leukocyte count between infected and noninfected children finding no significant differences (not shown). These results argued against a possible indirect role of these infections in the onset and the course of disease.

## 4. Discussion

Epidemiological data suggest the involvement of infections in the etiology of childhood ALL; however, the identity of the potentially implicated agent(s) has not been identified. Based on the original Smith's proposal about a direct causative role of viral agents in ALL development [7], we screened the presence of EBV, HCMV, HHV6, and HHV7 viral genomes in B-cell and T-cell ALL samples by a standard PCR aiming to match the number of infected cells with the number of leukemic blasts. The infectious etiology of childhood leukemia has been mainly proposed for common B-cell ALL [5, 7]. However, in this work, the viral screening in T-cell ALL was performed as an exploratory analysis based in the known T-cell tropism of the studied herpes viruses. Also, EBV is the causative agent of T-cell lymphomas [36, 37] and a role of EBV in the etiology of T-cell leukemia has been suggested [38].

We found no evidence of the participation of EBV, HCMV, HHV6, and HHV7 in the B-cell and T-cell ALL development. Positive samples were detected only through a more sensitive nested PCR, most likely reflecting normal levels of infection according to the age of the tested population. Positivity only by nested PCR does not support a relationship between infection and leukemogenesis. Still, the nested PCR was implemented as an additional control to globally validate our detection system. Based on the knowledge that the herpes viruses under study are ubiquitously acquired early in life and maintain a reservoir population in bone marrow, we expected to obtain positive samples by a sensitive detection, reflecting the common infection occurring in the pediatric population.

Previous studies looked for the presence of DNA viruses in bone marrow or peripheral blood from childhood ALL samples, namely, JVC, BKV, and SV40 polyomaviruses [39, 40]; TT anellovirus [41] and EBV and HHV-6, -7, and -8 herpes viruses [42]. Nevertheless all of them have failed to find evidence of their participation in the disease. Although, there is a previous work analyzing the participation of herpes viruses in leukemogenesis with similar negative results, based on the epidemiological differences between our country and developed countries related to the increased prevalence of infections and childhood leukemias in Mexico [1], we hypothesized that a mechanism of direct leukemogenesis could explain some cases of leukemia in our population. An advantage of our work over previous studies is the number of samples analyzed, *N* = 70 in our study versus *N* = 15–47 in others; which conferred it a higher statistical power. Also, other works screened the presence of viral genomes of EBV and HHV6 herpes viruses [43], JVC and BKV polyomaviruses [44], and parvovirus B19 [45] in archived neonatal blood spots collected at birth from children who developed leukemia, however, these works lost the window of infections happening from birth to the presentation of the disease. Additionally, this is the first work reporting no association between HCMV and childhood ALL. We have also previously reported that human T-cell lymphotropic virus type 1 (HTLV1) and mouse mammary tumor virus -like virus (MMTV-LV) were not involved in the genesis of childhood ALL [29].

Indirect mechanisms of leukemogenesis are also possible but addressing those mechanisms was beyond the aim of this study. Still, we did not find any evident association between nested PCR positive patients and clinical data. Previous studies have supported a role for EBV in genetic instability and EBV-associated Burkitt's lymphoma is characterized by a* Myc* genetic translocation [46–48]. However, the low number of patients with genetic translocations in our study (*N* = 8) precluded an analysis of EBV infection and ALL-associated genetic translocations with significant statistical power. It is proposed that herpes viruses often act in concert potentiating their pathological effects in cases of chronic fatigue syndrome, infectious mononucleosis, and/or posttransplant disease [31–35]. Although, we had a sizable number of infection positive patients, most of them were single infected, with only 6 (9%) showing evidence of infection by more than one herpes virus (see Table 3). Therefore, we do not have any evidence of a possible interaction by the tested viruses.

It is possible that the lymphoproliferation that characterizes ALL results from continuous immune stimulation triggered by repetitive infections. However, almost any infectious agent would be responsible for this effect and only tumor viruses with documented or possible direct transforming properties were tested here. There exist some examples of chronic antigenic stimulation leading to lymphomas, for instance, MALT lymphoma after* Helicobacter pylori* persistent infection and hepatitis C virus associated lymphoma [49–52]. Since those are adult neoplasias arising after decades of antigenic stimulation it is unlikely that pediatric ALL could result from a similar mechanism and arise as the immune system responds to a single infection. In any case, it is more likely that multiple antigenic challenges combined with maturing lymphocytes with a genetic lesion, for example, an intrauterine translocation, were involved in the genesis of ALL. In lymphomas of mature cells, the identity of the target of the antigen receptor (VDJ recombination clone) gives light to the origin of the lymphoproliferation. Since ALL occurs in progenitor cells in which more often the leukemic blasts have not completed the rearrangements of the antigen receptor, this is a scenario more difficult to corroborate.

An important limitation of all studies searching for infectious agents in pediatric ALL is that all have been performed individually, testing samples through standard techniques of molecular biology (PCR and/or Southern blot). Such strategies are restricted to agents whose genomic sequences have been at least partially described. Future investigations should use next-generation sequencing technologies and bioinformatic analysis that are more able to detect the full spectrum of viruses including those still not described, as it has been recently shown for Merkel cell polyomavirus [53]. Besides tumor viruses, indirect mechanisms of leukemogenesis also need to be addressed in order to better understand the role that a history of infection has in the development of pediatric ALL.

## Figures and Tables

**Table 1:** Demographic and clinic characteristics of the patients *N* = 70.

Age at diagnosis (years)	Median (range)
	7.6 (0.8–15.7)

Gender	*N* (%)
Male	33 (47)
Female	37 (53)

Birthplace	*N* (%)
Mexico City	63 (91)
Other Southern Mexican States	7 (9)

Percentage of blasts in bone marrow	Average (range)
	87% (25%–100%)

Immunophenotype∗	*N* (%)
B-cell precursor	66 (94.3)
T-cell	4 (5.7)

FAB classification	*N* (%)
L1	43 (62)
L2	26 (36)
L3	1 (2)

Genetic rearrangements∗∗	*N* (%)
ETV6-RUNX1	6 (12)
E2A-PBX1	2 (4)
No rearrangement	42 (84)

*Immunophenotype was determined according to international parameters [54].

∗∗Molecular diagnosis of ETV6-RUNX1, E2A-PBX1, MLL-AF4 and major and minor BCR-ABL genetic rearrangements were performed only in 50 samples. Percentages shown were calculated using 50 as denominator.

**Table 2:** PCR cycling conditions and primers sequences.

Virus/endogenous gene	Type of PCR	Cycling conditions^(a)^	Primers sequences (5′→3′)
EBV	Standard	95°C/40 sec, 57°C/1 min, 72°C/1.5 min (30*X*)	**F** CCATGTAAGCTTGCCTCGAG **R** GCCTTAGATCTGGCTCTTTG [55]^(c)^

EBV	Nested	95°C/20 sec, 57°C/30 sec, 72°C/45 sec (15*X*)	**F** CTTTGTCCAGATGTCAGGGG **R** GCCTGAGCCTCTACTTTTGG^(b)^

CMV	Standard	95°C/1 min, 55°C/1 min, 72°C/1 min (35*X*)	**F** AAGATGCGGTAGATGTCGTT **R** CTGCGCTCTTCTTTTTCGAT^(b)^

CMV	Nested	95°C/45 sec, 55°C/45 sec, 72°C/45 sec (15*X*)	**F** TTCTGACCCTGAACCGTAG **R** CGACGAAGAACTCGTAACC^(b)^

HHV6^(d)^	Standard	95°C/1 min, 50°C/1 min, 72°C/1 min (30*X*)	**F** GTGCGCTATAAAATCGATAGC **R** TGATTTCCGTTGTGTGTTTTCC^(b)^

HHV6^(d)^	Nested	95°C/30 sec, 56°C/30 sec, 72°C/30 sec (15*X*)	**F** GTCTCTTCGTATCCACGCG **R** CGTTCCCGTCGAAGAAATC^(b)^

HHV7	Standard	95°C/45 sec, 53°C/45 sec, 72°C/45 sec (30*X*)	**F** TTTTTACATTTGGCTTGCTTTTTG **R** ATATTTCTGTACCTATCTTCCCAA [56]^(c)^

HHV7	Nested	95°C/30 sec, 55°C/30 sec, 72°C/30 sec (15*X*)	**F** GAACGGTTTGCTTAGATTGC **R** GCAGACCAAACTCCACAAATTC^(b)^

*β*-actin	Standard	95°C/1 min, 60°C/1 min, 72°C/1.5 min (30*X*)	**F** CCTAAGGCCAACCGTGAAAAG **R** TCTTCATGGTGCTAGGAGCCA [57]^(c)^

^(a)^All amplification runs included an initial denaturation step at 95°C for 5 minutes and a final extension step at 72°C for 10 minutes. Annealing temperatures were optimized for every reaction.

^(b)^These primers were designed in our laboratory using the Primer-BLAST program [25].

^(c)^The specificity of the previously reported primers was corroborated using Primer-BLAST program [25].

^(d)^HHV6 primers recognize both HHV6A and HHV6B subtypes.

**Table 3:** Infection and prognostic factors.

Virus	EBV+	HCMV+	HHV6+	Any coinfection
*N* (%)	10 (14%)	13 (19%)	6 (9%)	6 (9%)
High-risk leukemia				
OR (95% IC)	0.43 (0.12–1.57)	0.72 (0.22–2.32)	2.29 (0.4–15.32)	1.12 (0.24–5.2)
*P*	0.29	0.75	0.4	1
Relapse				
OR (95% IC)	1.19 (0.27–5.36)	0.88 (0.20–3.82)	1.10 (0.15–6.63)	0.81 (0.12–4.67)
*P*	1	1	1	1
Death				
OR (95% IC)	0.17 (0.02–1.14)	0.16 (0.02–1.08)	0.04 (0.04–2.81)	0.37 (0.05–2.03)
*P*	0.89	0.08	0.65	0.41

OR: odds ratio; CI: confidence interval. *P* < 0.05 was considered significant.
